# The Role of Cyclosporine in the Treatment of Severe Ulcerative Colitis in the Pediatric Population: A Narrative Review Exploring Known Strategies and New Possibilities

**DOI:** 10.3390/jcm14238273

**Published:** 2025-11-21

**Authors:** Anna Dancewicz, Katarzyna Akutko, Hubert Paweł Szyller, Gabriela Augustynowicz, Tomasz Pytrus

**Affiliations:** 1 2nd Clinical Department of Paediatrics, Gastroenterology and Nutrition, Wroclaw Medical University, 50-369 Wroclaw, Poland; anna.dancewicz@umw.edu.pl (A.D.);; 2 Student Scientific Group of Pediatric Gastroenterology and Nutrition, Wroclaw Medical University, 50-369 Wroclaw, Poland

**Keywords:** inflammatory bowel disease, cyclosporine, ulcerative colitis, gastroenterology, pediatrics

## Abstract

Cyclosporine is a selective calcineurin inhibitor used, among other areas, in pediatric gastroenterology as rescue therapy in the treatment of severe ulcerative colitis (ASUC—acute severe ulcerative colitis) in children. The introduction of systemic glucocorticosteroids (GCSs) has significantly reduced mortality in ASUC. However, it should be emphasized that long-term use of these drugs is limited due to the high risk of adverse effects observed during therapy and their limited efficacy. The role of cyclosporine as a second-line therapy is referred to as a ‘rescue therapy,’ which aims to alleviate the symptoms of ASUC, often avoiding life-threatening complications (including toxic megacolon) and postponing the need for urgent colectomy. If colectomy is necessary, this provides time to better prepare the patient nutritionally and psychologically for surgery and to await the effect of slower-acting thiopurine preparations or other chronic treatments; their effectiveness in achieving long-term clinical and endoscopic remission is limited. New therapeutic approaches include cyclosporine as an inducer, which acts as a bridge to new forms of therapy, such as biological drugs, which are used as maintenance preparations in patients. In pediatric patients, there is limited research in the literature on new strategies for the use of cyclosporine. The aim of this review is to assess current evidence on cyclosporine as induction or rescue therapy in pediatric ASUC and explore future integration with biologic and biosimilar strategies, emphasizing its evolving role as a bridging agent toward biologics and novel targeted therapies.

## 1. Introduction

Ulcerative colitis (UC) is a chronic inflammatory disease of the gastrointestinal tract with mild, moderate, or severe symptoms. It affects the colon and rectum and most often manifests itself as chronic diarrhea, bleeding from the lower gastrointestinal tract, and abdominal pain [1].

In recent decades, there has been an increase in the incidence of UC, including among children. Data in the literature show that in this group of patients, the extent of inflammatory changes is often greater and the course of the disease is more severe compared to the adult population. As a result, UC is also a major therapeutic problem in pediatrics.

There is still a lack of data on the incidence of UC in the pediatric population. A review published in 2022 estimated that the incidence of inflammatory bowel disease (IBD) in children was highest in Germany, Sweden, Scotland, and Nova Scotia (Canada), and lowest in Saudi Arabia and Libya. The incidence per 100,000 people ranged from 5.0 to 52.2 in Asia, 31.0 to 75.0 in Europe, 28.3 to 63.6 in Canada, and 21.7 to 46.0 in Oceania. Crohn’s disease (CD) was observed twice as often in these populations compared to ulcerative colitis (UC). In the pediatric population with UC, compared to the adult population, the location of the disease has a wider range. Colitis is observed in 90% of cases, and proctitis is observed in 4 to 13% of cases. Isolated proctitis may occur in 30% of pediatric cases [2].

The etiology and pathogenesis of UC remain unclear. It is believed that environmental factors, as well as interactions between genetic predisposition, intestinal microflora, and the immune response of the intestinal mucosa, are responsible for the onset of UC [3,4]. It is worth noting that the increase in the frequency of UC diagnosis is also influenced by increased awareness among medical personnel and a reduction in the time from the first symptoms to diagnosis.

Acute severe UC (ASUC) is a potentially life-threatening condition in pediatric patients and requires urgent hospitalization. ASUC in the adult population is diagnosed based on the Truelove and Wits criteria, which include an assessment of the frequency of blood in the stool (6 or more times per day) and at least one of the following criteria: heart rate > 90 beats per minute, temperature > 37.8 °C, hemoglobin < 10.5 mg/dl, and ESR 30 mm/h [1,5]. In the pediatric population, the Pediatric Ulcerative Colitis Activity Index (PUCAI) is a more current and accurate scale, in which a score of ≥65 points is defined as ASUC. Clinical parameters are presented in Table 1.

Furthermore, the PUCAI scale is also used to assess the response to GCS (Glucocorticoids) used in the treatment of ASUC and to decide on a change in treatment [6]. Treatment options for pediatric and adult patients are similar. The first-line treatment for ASUC is intravenous steroid therapy (hydrocortisone/methylprednisolone), and if this is ineffective, biological drugs or calcineurin inhibitors (cyclosporine, tacrolimus) are used [7]. Intravenous steroid therapy achieves a response in 71% of cases [1]. However, despite therapeutic advances and the widespread use of biological drugs, the colectomy rate in the adult population remains high at around 30% [1]. A clinical problem is posed by patients who do not respond to first-line treatment—steroid-resistant patients or patients in whom it is not possible to discontinue steroid therapy without recurrence of symptoms or exacerbation within 3 months of the end of treatment—including steroid-dependent patients [8]. The overall rate of steroid resistance in ASUC in the pediatric population is higher than in the adult population (34% vs. 29%) [9,10].

Due to the increasing number of pediatric patients with ASUC who have failed previous attempts at biological therapy, there is a need to develop new strategies for treating such patients. This review presents the mechanism of action of cyclosporine as one of the drugs used in the treatment of ASUC. In addition, based on the available literature, various therapeutic strategies using cyclosporine were analyzed, and their effectiveness was evaluated with regard to the pediatric population.

## 2. Materials and Methods

A search of PubMed and Web of Science databases was conducted in order to identify all articles published up to 30 September 2025, based on the following keywords: “cyclosporine” or “ciclosporin,” “pediatrics” or children” or “kids,” and “colitis ulcerosa” or “ulcerative colitis”, which are included in this narrative review. Review articles on ASUC were meticulously checked in person for additional studies to ensure that no relevant work was overlooked. The study included all currently available online original works on the effects of treating the pediatric population with ASUC with cyclosporine preparations.

The search for articles was limited to English-language publications from 1990 to 2024.

This review included studies that met the following criteria:

- Studies conducted in children and adolescents with severe ulcerative colitis (0–18 years of age);
- Treatment with cyclosporine;
- Studies written in English.

Studies were excluded according to the following criteria:

- Studies conducted in adults and rejecting articles containing mixed populations (adults and children) due to the inability to distinguish potential differences between these two patient groups;
- Case series with fewer than five patients;
- Studies that were letters or editorials;
- Studies that lacked sufficient raw data;
- Studies that were duplicated;
- Studies that were ongoing or incomplete;
- Studies for which only an abstract was available.

The parameters of this review were as follows:

- Short-term clinical response lasting 2–9 days;
- The need for urgent surgical intervention within one month of treatment with cyclosporine;
- Long-term clinical response lasting more than 6 months;
- The need for colectomy in the study group as a whole;
- Reported adverse effects of cyclosporine therapy.

**Figure 1:**
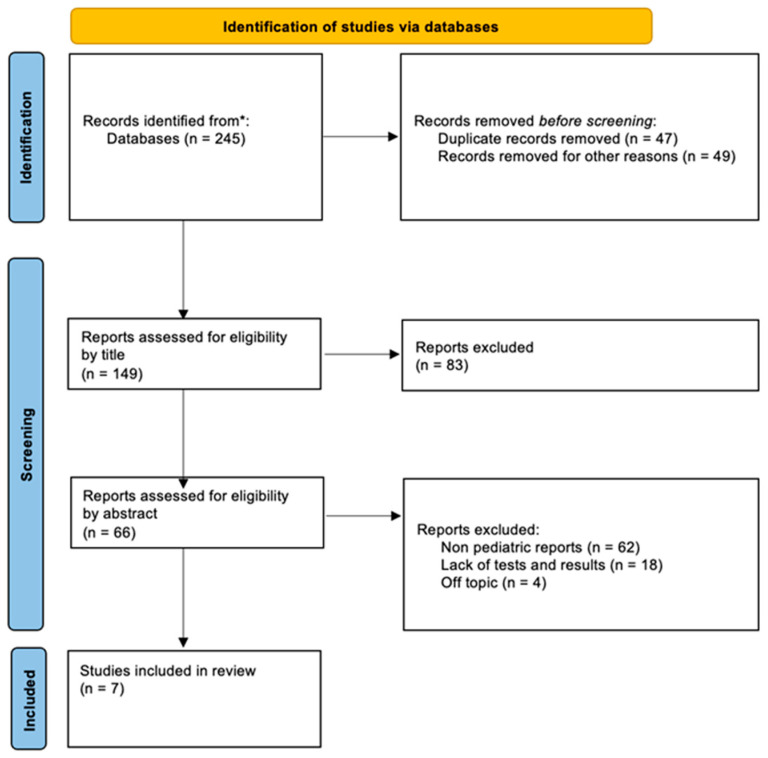
Flow diagram of documents found in the PubMed and Web of Science databases included in this article. * PubMed and Web of Science databases.

A diagram depicting the process of selecting articles included in the review is presented in Figure 1.

## 3. Cyclosporine—Mechanism of Pharmacological Activity

Cyclosporine A is a calcineurin inhibitor that selectively blocks T-cell immunity. It is a cyclic, hydrophobic peptide consisting of 11 amino acids.

Cyclosporine was isolated in 1972 during research on antifungal compounds from the fungus *Tolypocladium inflatum* [11]. Its immunosuppressive effect was discovered only after 4 years, as it was initially thought to have only limited antifungal activity. In the early years, it was used as a drug to prevent transplant rejection, and in subsequent years, its effectiveness was also observed in the treatment of other diseases.

The mechanism of action of cyclosporine is based on selective blocking of T-cell immunity. Cyclosporine forms a complex with the protein cyclophilin (a cytoplasmic protein of T lymphocytes). This complex inhibits the activity of calcineurin (cytoplasmic serotonin–threonine phosphatase). Inhibition of calcineurin prevents dephosphorylation and translocation to the cell nucleus of the nuclear factor of activated T cells. As a result, it reduces the expression of the IL-2 gene. T lymphocytes do not produce IL-2, which is a cytokine necessary for the full activation of T lymphocytes, as well as interferon gamma and granulocyte-macrophage colony-stimulating factors. As a result, cyclosporine leads to the inhibition of T cell proliferation and differentiation towards cytotoxic lymphocytes, inhibits humoral and cellular immune responses, modifies chronic inflammatory processes, and also removes macrophages present in the epidermis and skin and inhibits the release of histamine from mast cells [12,13]. Cyclosporine is biotransformed mainly in the liver and, to a lesser extent, in the gastric mucosa, mainly with the participation of cytochrome P-450 isoenzyme CYP3A4 and CYP3A5 [14]. Therefore, it is necessary to collect accurate information about the medications used by the patient, as drugs that inhibit or stimulate the cytochrome P450 system may affect the concentration of the drug. The rate of cyclosporine excretion in children is up to 4 times higher than in adults over 40 years of age. This is probably due to the shorter length of the intestine rather than differences in drug metabolism [15]. The use of cyclosporine is limited by the side effects of the therapy. Contraindications to the use of the drug include uncontrolled hypertension, severe renal failure, severe infections, and a positive history of cancer. Common side effects in adult patients after using cyclosporine preparations include hirsutism, tremors, headaches, hypertension, hyperlipidemia, and renal dysfunction. Particularly dangerous and requiring vigilance are the adverse effects of the drug in the form of nephrotoxicity, causing a decrease in glomerular filtration, damage to the renal interstitium, and macroangiopathy of the renal arteries [16,17]. For this reason, patients taking cyclosporine preparations should have their serum creatinine levels monitored, and if an increase of at least 30% is observed in two measurements taken every two weeks, the dose should be reduced. If the creatinine concentration does not decrease despite the dose reduction, this is an indication to discontinue therapy [16].

In the pediatric population, gingival hyperplasia is a statistically common adverse effect of cyclosporine therapy. According to some sources, it occurs in up to 30% of pediatric patients. Gingival hyperplasia most often occurs within the first 3–6 months of treatment [18,19]. This is confirmed by a study conducted by Socha et al., in which adverse effects were observed in 2/23 children included in the study—these were hirsutism and gingival hyperplasia [20]. In summary, during treatment with cyclosporine, it is recommended to monitor the concentration of the drug in the blood, but also to monitor: liver function, lipid profile, serum creatinine, urea, uric acid, and electrolyte levels (especially magnesium and potassium), blood pressure (before starting treatment, after 2, 4, 6, and 8 weeks) and blood glucose levels.

Mild side effects during cyclosporine use are quite common but do not limit therapy, while severe side effects are rare but significantly limit the use of cyclosporine in ASUC [1].

Modern strategies aimed at reducing cyclosporine A (CsA) nephrotoxicity increasingly focus on the use of nanocarriers and targeted drug-delivery systems. Ankola et al. demonstrated that biodegradable nanoparticles loaded with CsA achieved a Cₘₐₓ comparable to the conventional formulation (Neoral^®^) while producing significantly lower markers of renal injury (reduced serum urea, creatinine, and glomerular damage) in an animal model, an effect attributed to a delayed Tₘₐₓ and sustained drug release from the carrier [18]. Moreover, nanocarriers such as PLGA-based nanoparticles or nanosuspensions (e.g., CsA nanosuspension improving bioavailability while reducing renal accumulation) have been developed to minimize dosage requirements and nephrotoxic exposure [19]. In a recent review, Aljabri et al. emphasized that similar advanced drug delivery platforms, originally designed for biologic therapies, may also enhance the safety of immunosuppressive agents by improving biodistribution and mitigating systemic adverse effects, including nephrotoxicity [21]. Considering the multifactorial mechanisms of CsA-induced renal injury, such as afferent arteriolar vasoconstriction, reduced renal blood flow (RBF), activation of TGF-β1, mTOR, and NF-κB pathways, and interstitial fibrosis, such as pharmacokinetic modulation appears crucial [22,23]. Through precise tissue targeting and reduced renal accumulation, nanocarrier-based systems may enable lower effective doses, decreased renal cellular toxicity, and an overall improved therapeutic index of CsA.

## 4. The Role of Cyclosporine in the Treatment of Ulcerative Colitis in Pediatrics—Previous Strategy

According to the 2018 guidelines of the European Crohn’s and Colitis Organization and the European Society of Paediatric Gastroenterology, Hepatology and Nutrition (ESPGHAN), cyclosporine may be considered as an alternative second-line therapy when steroid therapy is ineffective in the treatment of ASUC in children.

However, infliximab is recommended as a second-line treatment in children who have not previously received TNF-blocking therapy and in whom intravenous steroid therapy has failed [6].

The action of cyclosporine appears to be well understood, and its role in the therapeutic regimen is well established, but also highly limited. Data presented in a pediatric review show that infliximab and cyclosporine are equally effective in inducing a short-term clinical response in ASUC in the pediatric population (77% vs. 81%). However, they differ with a clear advantage for infliximab in long-term management (64% for infliximab compared to 39% for cyclosporine) (*p* < 0.001) [10]. Additionally, the use of infliximab is supported by good and long clinical experience in many centers, a better risk-benefit profile of this preparation, and the possibility of continuing maintenance treatment [6]. The role of cyclosporine as a fast-acting calcineurin inhibitor has been limited to selected cases, such as induction therapy and then as bridging therapy to other long-term maintenance treatment with thiopurines. In addition, its relatively high toxicity profile makes it optimal to discontinue its use once thiopurines become effective, usually after 3–4 months [10]. Therefore, among steroid-resistant patients who have failed previous thiopurine maintenance therapy, infliximab is again the preferred second-line therapy. Cyclosporine was introduced into the treatment of steroid-resistant patients in 1994—Lichtiger et al [24]. concluded on the basis of their studies that intravenous cyclosporine is effective in patients with severe ulcerative colitis resistant to corticosteroids [25]. Lichtiger et al. [24] observed that 9/11 (82%) of adult patients with severe steroid-resistant UC responded (within a short time) to treatment with oral or intravenous cyclosporine at a dose of 4 mg/kg/day c, while all 9 receiving placebo showed no improvement. This was a placebo-controlled, multicenter study that also showed that two-thirds of patients experienced long-term benefits from cyclosporine treatment.

Table 2 summarizes the available studies on the use of cyclosporine in ASUC in the pediatric population. The data presented in the table are divided into assessment of short-term and long-term clinical response to cyclosporine treatment, the need for urgent surgical intervention, the need for colectomy during the entire clinical observation in the study group, and adverse events observed during cyclosporine therapy.

In a study published in 2006 by Castro et al. [28], which aimed to evaluate the efficacy of oral cyclosporine treatment in the pediatric population, 32 pediatric patients with refractory or glucocorticoid-dependent ASUC were included in the clinical observation. These patients were administered oral cyclosporine under the control of its blood concentration (150–250 ng/mL). The results of this study showed the high efficacy of oral cyclosporine, which prevented or delayed the need for colectomy with minimal side effects of this therapy. Twenty-eight patients (87.5%) responded quickly to treatment, and only 4 patients (12.5%) required urgent colectomy. In summary, 9 surgical procedures were performed, which means that 72% of pediatric patients avoided colectomy. This confirms that cyclosporine, in many cases, allows urgent colectomy to be avoided and allows for planned preparation of the patient for surgery. In addition, in the vast majority of patients (26/28) who responded to cyclosporine treatment, it was possible to discontinue or reduce the dose of corticosteroids. Oral administration of cyclosporine minimized side effects [28].

A retrospective study conducted in a group of Italian children also demonstrated the high efficacy of cyclosporine in short-term treatment and in avoiding urgent colectomy. In a group of 16 patients with ASUC treated with intravenous cyclosporine, 2 patients underwent urgent colectomy within 10 days, and in another 2, treatment was discontinued due to a severe reaction after treatment. Twelve patients avoided urgent colectomy, while 11 patients required colectomy in the long term.

### Cyclosporine as a Means of Reducing the Necessity for Surgical Treatment

Cyclosporine plays an important role in preventing urgent surgical intervention—it allows time for intervention and psychological preparation of the patient for surgery. However, it is not an effective long-term treatment. Its safety profile in long-term therapy, especially when intravenous administration is necessary, is not satisfactory. In this group of patients, adverse effects were observed in 6 out of 23 patients (26%), and in three (13%), serious adverse effects were observed, due to which cyclosporine had to be discontinued. It is worth noting that all patients who developed an episode of neurotoxicity received cyclosporine intravenously [28].

Socha et al. [20] also conducted a study on the efficacy and safety of cyclosporine in children with IBD. The study was retrospective in nature. The study included 23 patients, including 21 with UC and 2 with CD, aged 2–18 years, who had been using cyclosporine preparations for 5 years. Cyclosporine preparations were administered in steroid-resistant exacerbations of the disease (in 10 patients) and in cases of steroid dependence (in 13 patients). Two patients experienced adverse effects in the form of gingival hyperplasia and hirsutism. In addition, only short-term (less than 6 months) clinical improvement was observed in 11 patients with UC. It is worth noting that all 10 patients with ASUC required colectomy. In 4 of the 10 patients with ASUC, this was a planned colectomy, so the use of cyclosporine in these cases allowed for better preparation of patients for surgery. In this group of patients, although cyclosporine was shown to be a safe drug (only 9% of patients experienced adverse effects), its effectiveness in achieving long-term remission in patients with ASUC was low [29].

Treem et al. [26] reported that 11 of 14 patients (78%) responded within two to nine days. However, only 4 of 14 patients (28%) avoided colectomy during a follow-up period ranging from 6 months to 5 years. Most patients who initially responded to treatment required colectomy due to disease exacerbation, and cyclosporine therapy served to better prepare the patient for surgery—rapid symptom relief, prevention of emergency colectomy, improved nutrition, psychological adaptation, and reduction in steroid dosage [26].

Another noteworthy study in the pediatric population is the study conducted by Ramakrishna et al. [25], evaluating the efficacy of cyclosporine in combination with azathioprine (or 6-mercaptopurine) in steroid-resistant patients. The aim of the study was to evaluate the possibility of achieving remission during bridging therapy with cyclosporine, followed by the use of azathioprine/6-mercaptopurine as the sole immunosuppressive agent. Six patients with steroid-resistant ulcerative colitis and two patients with Crohn’s disease received 100–200 micrograms/kg/day of cyclosporine intravenously, followed by 4–10 mg/kg/day orally. Seven out of eight patients responded quickly, and four of them did not undergo surgery. No significant complications of treatment were observed. On this basis, it was demonstrated that combination therapy with cyclosporine and azathioprine or 6-mercaptopurine can be safely used to maintain cyclosporine-induced remission in children with IBD [25]. In summary, data on short-term and long-term clinical response to cyclosporine treatment in the adult population are similar to those in the pediatric population.

In a 2023 review, the average short-term response rate (defined in various ways, but most often as avoidance of colectomy) in the adult population was calculated to be 70% (weighted average, 95% confidence interval [CI] 68 to 72%). The long-term response rate is relatively high at 52% [weighted mean; 95% CI 50 to 54%] [1]. In a similar review of the pediatric population, the short-term efficacy of cyclosporine as rescue therapy in steroid-resistant children with UC was 81% (95% CI: 76–86%), but only 39% (29–49%) in the long term [10]. The long-term success rate was higher in two studies in which immunomodulators were introduced at discharge to all patients (combined rate 71% (55–83%)) [25,28].

According to the 2018 pediatric consensus recommendations, patients who fail to achieve remission after second-line therapy should be referred for colectomy. However, in highly specialized centers and in selected non-urgent cases, it is acceptable to use sequential therapy with calcineurin inhibitors after infliximab or vice versa (after discontinuation of steroid therapy) [6].

No information on the use and evaluation of the efficacy of third-line sequential therapy in the pediatric population was found in the literature. Data on such treatment in the adult population are very limited, and the authors of these studies draw particular attention to the low efficacy of such treatment, the high risk of adverse effects, and the need for particular caution when using this approach. In the cited retrospective study of 19 patients with ASUC who received infliximab after cyclosporine failure (10 patients) or cyclosporine after infliximab failure (9 patients), remission was achieved in only about one-third of patients (40% vs. 33%), and remission was of limited duration (10.4 months vs. 28.5 months). In addition, serious adverse events occurred in 16% of patients, including 1 death, indicating that the risks associated with rescue therapy may outweigh the benefits [30]. Similar data can be found in a retrospective study conducted between 2000 and 2009, which evaluated a group of 86 patients who were treated sequentially with cyclosporine and infliximab. During the study, 49 patients did not respond to second-line rescue therapy and underwent colectomy [31].

## 5. New Strategy for the Use of Cyclosporine as an Induction Medication

Cyclosporine is a fast-acting immunosuppressive drug, the efficacy and role of which in ASUC therapy has been confirmed in the scientific studies cited above. Cyclosporine can be used as rapid induction therapy in patients with contraindications to steroid therapy or in cases of steroid resistance, as well as in patients with prior exposure, lack of response, or contraindications to anti-tumor necrosis factor (TNF) therapy. The decision on the further treatment strategy and transition from cyclosporine treatment to a long-term maintenance agent is necessary before starting therapy. The intensive development of biological therapies in recent years has brought hope for a change in strategy and new opportunities to exploit the potential of cyclosporine, which has been used less and less frequently in many centers. The new approach is based on the use of cyclosporine as an induction agent for new drugs such as vedolizumab and usterkinumab [32].

Currently, due to the greater exposure of patients to thiopurines and anti-TNF drugs, the development of new forms of treatment requires well-planned studies to evaluate the effectiveness of cyclosporine in UC as a bridge to maintenance therapy with vedolizumab, ustekinumab, or other forms of therapy. The following section cites studies on the efficacy of cyclosporine as a bridge to other forms of therapy, primarily in the adult population, due to the lack of studies in the pediatric population. Studies on the efficacy of cyclosporine as induction therapy in the adult population give hope for the use of these new forms of therapy in the pediatric population as well.

### 5.1. Vedolizumab

Vedolizumab is a humanized IgG1 monoclonal antibody that binds to human integrin α4β7, produced by Chinese hamster ovary (CHO) cells. Guidelines suggested that vedolizumab could be used to treat UC in which anti-TNF therapy had failed [33], as well as UC resistant to steroids or anti-TNF [34]. In the pediatric population, the European Crohn’s and Colitis Organization (ECCO) and the European Society for Pediatric Gastroenterology, The European Society for Paediatric Gastroenterology, Hepatology, and Nutrition (ESPGHAN) recommended the use of vedolizumab monotherapy for UC in chronically active or steroid-dependent patients as second-line biologic therapy after failure of anti-TNF therapy [35]. Vedolizumab is an effective drug for inducing and maintaining remission. Due to its local action, it has a very favorable safety profile [32,33,36] In a study by Colombel et al [37]., vedolizumab was shown to have a favorable safety profile, with a low incidence of serious infections, infusion-related reactions, and neoplasms during long-term treatment. Exposure to vedolizumab was not associated with an increased risk of serious infection. Severe Clostridium difficile infections, sepsis, and tuberculosis were rarely observed (≤0.6% of patients) [37]. Due to its better safety profile compared to other forms of therapy, vedolizumab appears to be a promising biological drug in the pediatric population. A limitation in the use of vedolizumab is the longer time to reach its full effect [it acts gradually and reaches full efficacy in 6–8 weeks] [34,38]. The combination of vedolizumab with a fast-acting drug such as cyclosporine appears to be the optimal form of therapy for patients with UC, especially for patients who cannot tolerate other conventional drugs or have developed adverse reactions to them [39]. No data on the use of combination therapy with cyclosporine and vedolizumab in the pediatric population were found among the articles analyzed. All available studies in the pediatric population are based solely on the use of vedolizumab as monotherapy and do not involve patients with UC. The only available publication found was a letter to the editor from an Italian center in 2021 describing the case of a teenage patient with UC complicated by pyoderma gangrenosum, who had previously been treated with thiopurines, infliximab, and adalimumab for various reasons without effect. After successful induction, vedolizumab was used as maintenance therapy. During this therapy, the patient developed skin ulceration on the tibia, diagnosed as pyoderma gangrenosum, with no gastrointestinal symptoms. It is worth noting that pyoderma gangrenosum is more common in patients with active colitis. In this case, oral immunosuppressive therapy with cyclosporine was initiated, achieving clinical remission, and the skin lesion was also in remission [40]. However, this is not a case in which the role of cyclosporine would be of interest to us in this review: remission induction and bridging therapy for further treatment with vedolizumab. However, it is worth citing data from a study by Gisbert et al. [1], which collected 8 studies involving a total of 156 patients and used this group of patients to evaluate the efficacy of vedolizumab in ASUC. The vast majority of patients had been exposed to anti-TNF drugs in the past, and studies using a true bridging strategy were included, i.e., cyclosporine and vedolizumab were included in the treatment during the same hospitalization. The overall response rate to vedolizumab (assessed as avoidance of colectomy) was 65% and was similar to the response rate in studies in which the patient received cyclosporine and vedolizumab not during a single hospitalization (69% weighted average; 95% CI from 61% to 76%) [1]. In a study by Ollech et al. [41], the authors presented the results of the efficacy of combined induction therapy with a calcineurin inhibitor [cyclosporine or tacrolimus], followed by maintenance therapy with vedolizumab. This study was conducted in a group of 71 adult patients with ASUC, and the observation period was 2 years. The survival rates without colectomy were 93% after 3 months, 67% after 1 year, and 55% after 2 years [41]. The second largest group of patients was included in the treatment by Pellet et al. [34], who collected data on 39 patients with active steroid-resistant UC (31 patients had active severe UC, and in 36, anti-TNF treatment was ineffective), who received a calcineurin inhibitor as induction therapy along with vedolizumab as maintenance therapy. After 1 year, 68% of patients survived without colectomy, with efficacy similar to the results of Ollech et al. [41]. However, when only patients with ASUC were evaluated, the annual colectomy rate was 36% [34]. In summary, the undoubted advantage of this form of therapy is its favorable safety profile, which may be a huge advantage when used in the pediatric population with ASUC who have previously failed anti-TNF or thiopurine therapy. Nevertheless, there is still a lack of studies comparing this new bridging strategy [with cyclosporine and vedolizumab] with conventional treatment.

### 5.2. Ustekinumab

With the more severe course of ASUC in patients and the greater availability of biological treatment, there is a growing group of patients in whom previous treatment with anti-TNF, thiopurine, and vedolizumab has failed. An increasing number of cases presented in the adult population indicate that combined treatment with cyclosporine and ustekinumab is a potential treatment option for patients in whom previous therapies have been ineffective. Ustekinumab, an IL-12/IL-23 antagonist, is characterized by high efficacy and a good safety profile when used, as shown in the studies cited in the table below [42,43,44,45,46] Studies evaluating the efficacy of cyclosporine and ustekinumab in the treatment of ASUC are summarized in Table 3 and include four retrospective studies. It is worth noting that the data published to date are based on descriptions of individual cases of patients with ASUC (the largest group included 11 patients) who received induction with cyclosporine and then continued maintenance treatment with ustekinumab. The total number of patients in the cited studies is 24. A study published by Ganzleben et al. [42] presented the case of a patient with steroid-resistant ASUC who had previously shown no response to anti-TNF therapy, vedolizumab, cyclosporine, thiopurines, and tofacitinib. It was decided to re-initiate induction therapy with cyclosporine, and after achieving an initial clinical response, ustekinumab therapy was administered. After 3 months of combination therapy (cyclosporine and ustekinumab), following clinical and endoscopic remission, cyclosporine was discontinued. The patient remained in remission throughout the 6-month follow-up period [42]. Another study from 2021 was conducted on a group of 2 patients with ASUC who had previously failed to respond to other therapies and were eligible for induction therapy with cyclosporine, followed by maintenance therapy with ustekinumab. Both patients avoided colectomy, and no serious adverse events were observed [42]. Similar data were presented in a study published by Veyrard et al. in 2022 [43]. This study included a group of 10 individuals who had also been treated with anti-TNF drugs and vedolizumab in the past. Patients who initially did not respond to calcineurin inhibitors or required urgent colectomy were excluded from this study. Thanks to this strategy, colectomy was avoided in all patients. One patient experienced an episode of alopecia areata, but this was considered a mild complication and treatment was continued [43]. The advantage of the study published by Vitali F et al. [44] over the previous studies cited is longer patient follow-up (one year), the largest group of patients included in the study (11 steroid-resistant patients, most of whom had been unsuccessfully treated with infliximab, vedolizumab, and tofacitinib), as well as the baseline clinical condition of the patients (the patients had a disease duration of over 9 years, high disease activity at the start of the study with a mean Mayo score of 10.9 and an endoscopic Mayo score of 2.7) [44]. Patients were systematically assessed in detail according to the Mayo score at each visit. During the observation period, a series of laboratory and ultrasound tests were performed, as well as endoscopic and histological evaluations, before inclusion in the study at 16 and 52 weeks of treatment. Patients received induction therapy with intravenous cyclosporine, which was then switched to oral form and continued for up to 6 months. Intravenous ustekinumab therapy was started on average 3.2 [1,2,3,4,5,6,7] weeks after the start of effective intravenous induction therapy with cyclosporine and continued in oral form. A clinical response was achieved in more than half of the treated patients—6 out of 11—at week 16 and was maintained until the end of the observation period at week 52. Clinical remission according to the Mayo score was achieved in five patients at week 48, and another patient achieved clinical remission but did not undergo additional endoscopic examination due to pregnancy. Endoscopic remission was also achieved in five patients at weeks 16 and 52, respectively. Among treatment failures, two patients had to undergo colectomy, and three patients discontinued ustekinumab and switched to other therapies during the 1-year follow-up period [44]. In summary, the use of cyclosporine in combination with ustekinumab may be considered in selected patients, especially those who have previously failed treatment with thiopurines, anti-TNF agents, or vedolizumab. There is a need for similar studies in the pediatric population, particularly among adolescents with ASUC. The reported studies are summarized in Table 3.

### 5.3. Tofacitinib

Tofacitinib is an orally administered, small-molecule, fast-acting inhibitor—a selective and reversible inhibitor of Janus kinases JAK1, JAK2, and JAK3, as well as TyK2. This drug appears to be an alternative for induction and maintenance therapy in moderate to severe UC due to its specific biochemical characteristics. Tofacitinib is rapidly absorbed and reaches its maximum serum concentration after approximately one hour. It can cause rapid clinical improvement as early as the third day of oral therapy [47].

Nevertheless, it is worth noting that the time to respond is individual and may vary from a few days to several months [48]. In addition, it is also rapidly eliminated from plasma, which minimizes complications in the event of an urgent colectomy [49]. Another biochemical feature of tofacitinib that speaks in its favor is the fact that it is a small molecule and is therefore less susceptible to loss associated with hypoalbuminemia and loss in the inflamed colon than other biological therapies [48]. To date, several studies have been published in the adult population showing that this drug may be effective as monotherapy in the treatment of acute severe UC in patients who have previously been exposed to infliximab [50] and may also be used alone as a second-line rescue therapy with promising results [51]. One study by Yang et al. [52] assessed the use of a combination of tofacitinib and cyclosporine in the treatment of refractory ulcerative colitis in a patient with primary non-response to infliximab. The study describes the case of a 45-year-old female patient previously treated with mesalazine who experienced steroid-resistant exacerbation of UC. The patient received steroid therapy for 7 days, followed by an accelerated induction regimen of intravenous infliximab at a dose of 300 mg. In the absence of clinical and endoscopic improvement, the treatment was considered ineffective, and it was decided to add intravenous cyclosporine at a dose of 3 mg/kg/day and oral tofacitinib (10 mg, twice daily). After 10 days, gradual clinical improvement was observed in the patient. After 6 months of combination therapy, oral cyclosporine treatment was discontinued and combination therapy with tofacitinib (5 mg twice daily) and mesalazine (1.5 g twice daily) was initiated [52]. This is an interesting case report, but it does not provide a basis for making recommendations. Furthermore, in light of other studies, tofacitinib monotherapy appears to be sufficient to induce and maintain remission in this indication and is a potentially safer option compared to combination therapy [50,51].

Large randomized, placebo-controlled studies are needed to determine whether tofacitinib has the potential to replace cyclosporine or infliximab in the second-line treatment of steroid-resistant ASUC in children as well.

### 5.4. Upadacitinib

No studies were found among the searched articles that evaluated the efficacy of cyclosporine as a bridge to alternative advanced drug therapy with upadacitinib, an oral selective inhibitor (belonging to the group of Janus kinase (JAK) inhibitors), in the pediatric and adult populations. The drug is a reversible, selective inhibitor of JAK1 kinase.

Upadacitinib is highly effective in inducing and maintaining remission in moderate to severe UC in clinical trials in the adult population [53]. The drug induces rapid clinical remission with high efficacy and a favorable safety profile. A study conducted in an adult population by R. Gilmore in 2023 [54] evaluated the clinical response in 6 patients after administration of upadacitinib at a dose of 45 mg, who had previously been treated with infliximab, and assessed their general condition on day 7 of treatment and the need for colectomy up to 16 weeks after the start of treatment. Five patients showed a response according to the established criteria by day 5 after admission, and 6 responded by day 7. At week 16, five patients did not require colectomy, and one underwent an uncomplicated colectomy at week 15 [54]. It appears that the rapid onset of action of upadacitinib and its favorable safety profile will significantly reduce the need for cyclosporine as an adjunct.

## 6. Potential Differences and Therapeutic Challenges in the Pediatric Population

### 6.1. Limited Evidence Base and Pharmacokinetic Differences

Although cyclosporine A (CsA)-based therapeutic regimens have been widely used in the treatment of inflammatory and autoimmune diseases, their safety and efficacy in the pediatric population remain insufficiently characterized. Most clinical data originate from studies conducted in adults, while pediatric literature largely relies on small retrospective series or isolated clinical case reports [55]. Children differ from adults in terms of pharmacokinetics—they exhibit distinct drug metabolism profiles dependent on the maturation of enzymatic systems (particularly cytochrome P450, especially CYP3A4/5), smaller distribution volumes, and altered renal elimination. These differences may contribute to greater variability in CsA concentrations and instability of the clinical response [21]. An additional concern is long-term toxicity in children treated with cyclosporine, chronic kidney injury, interstitial fibrosis, and tubular dysfunction have been reported even at therapeutic serum levels [56]. Due to the ongoing development of organs and the immaturity of the immune system, children may be more susceptible to both toxic effects and immunosuppression, warranting caution when extrapolating data from adult studies.

### 6.2. Limitations and Controversies Related to Current Therapies

The use of CsA is associated with multiple limitations that must be considered in both pediatric and adult populations. The most relevant include substantial interindividual pharmacokinetic variability, a narrow therapeutic index, and the risk of renal drug accumulation leading to chronic nephropathy. Long-term CsA therapy, although effective in controlling disease activity, may cause hepatotoxicity, hypertension, lipid metabolism disturbances, and nephrotoxicity [49,56]. Some analyses have also highlighted a decline in clinical efficacy after prolonged therapy, likely related to immune adaptation and tolerance mechanisms [57]. Furthermore, emerging evidence suggests that the benefits of CsA may be inferior to those achieved with newer targeted or combined immunomodulatory strategies [58]. Therefore, ongoing safety monitoring and the development of innovative drug delivery systems, including nanocarriers and targeted formulations, are essential to minimize renal exposure and improve the therapeutic safety profile of CsA.

## 7. Conclusions

The traditional approach to cyclosporine as a second-line rescue therapy has significantly limited its use. Most centers have opted for anti-TNF therapy due to better experience with its use. The evolution of cyclosporine as induction therapy and the use of vedolizumab or usterkinumab as maintenance therapy opens up new therapeutic possibilities and offers new treatment options for particularly complex patients in whom previous treatment has proven ineffective. It is also worth mentioning the financial burden incurred by medical facilities in connection with the use of biological treatment, which could be reduced by a better understanding of the use of cyclosporine as an induction method.

The priority for the pediatric population is to establish treatment standards and regimens similar to those described above for adult patients, while also being aware of possible differences in therapeutic efficacy and complications, as well as potential methods for personalizing individual patient therapy based on differences in response, the remission achieved and disease severity.

## Figures and Tables

**Table 1:** Pediatric Ulcerative Colitis Activity Index (PUCAI). The PUCAI is a noninvasive tool assessing ulcerative colitis activity in children. Scores range from 0 to 85: <10 indicates remission; 10–34 mild; 35–64 moderate; and ≥65 severe disease.

Symptom	Description	Score
**Abdominal pain**	No pain	0
	Pain can be ignored	5
	Pain cannot be ignored	10
**Rectal bleeding**	None	0
	Small amount, <50% of stools	10
	Small amount with most stools	20
	Large amount (>50% of stool content)	30
**Stool consistency of most stools**	Formed	0
	Partially formed	5
	Completely unformed	10
**Number of stools per 24 h**	0–2	0
	3–5	5
	6–8	10
	>8	15
**Nocturnal stools (any episode causing wakening)**	No	0
	Yes	10
**Activity level**	No limitation of activity	0
	Occasional limitation	5
	Severe restriction of activity	10

**Table 2:** Summary of the articles described above, taking into account age, number of patients, and the effect achieved.

Author, Year	Type of Study	No. of Patients with UC and Sex	Age	Treatment in an Interview	Dose of Cyclosporine	Short Term Response (2–9 Days)	Short Time to Surgery (Less than 1 Month)	Long Term Efect (More than 6 Month)	Surgery Overall	Toxicity
J. Ramakrishna et al. [25]	observational retrospective	6	3–17 years	-	2 mg/kg/d iv. and then po. increased to blood level of 100–200 ng/mL cyclosporine,	83% (5/6 patients)		67% 4/6 patients (follow up 2–5 years)	67%	Tremor (*n* = 2), hirsutism (*n* = 1)
W R Treem et al. [26]	observational retrospective	14 (8 males and 6 females)	7–20 years	methylprednisolone iv. (1–2 mg/kg/day)	oral cyclosporine (4.6–9.6 mg/kg/day), cyclosporine blood levels 150–300 ng/mL.	78% 11/ 14 patients responded within 2–9 days.	21% 1/14 surgery 19 days after initiating therapy, 2/14 have elected surgery after 20 days of therapy and a partial response.	4/14 had recurrent symptoms after 2 to 11 months of taking therapeutic doses of cyclosporine and 3/14 flare ups while weaning from cyclosporine after 4 to 8 months. 3/14 patients have been weaned from cyclosporine after 8 to 13 months and have remained in remission from six months to five years. 1/14 complete a six-month course of cyclosporine.	72% 10/14 patients have undergone surgery.	
A. Barabino [27]	retrospective single center	16 (of 23)	4–13 years	12/16 azathioprine and 1/16 methotrexate, steroids iv., total parenteral nutrition, intravenous steroids and broad-spectrum antibiotics.	14/16 patients were treated iv. cyclosporine(12 on drip, four by bolus) at a mean daily dose of 3.2 ± 1.2 mg/kg (0.7–5 mg/kg), for a mean time of 10 ± 7 days (1–24 days), 2/16 patients were treated po. at a mean daily dose of 4.9 ± 1.1 mg/kg (3–7 mg/kg), for a mean time of 133 days (17–660 days)	75% 12/16 patients avoided urgent colectomy.	12,5% 2/ 16 patients underwent urgent surgery at a mean time of 10 days	6/16 patients underwent surgery at a mean time of 4.4 months: 4 on cyclosporine for unresponsive disease and 2 for relapse after drug discontinuation. 5/16 maintained remission (2on cyclosporine) at a mean time of 17 months. 1/16 had a partial response and discontinued the drug after a period of 2 months.	50% 8/16 patients have undergone surgery.	Headache (*n* = 2) seizures (*n* = 1), hypertension (*n* = 2), paresthesia (*n* = 1)
M. Castro [28]	observational retrospective	32 (17 females and 15 males)	22 months–18.5 years	32/32 continued treatment with 5-amino-salicylic-acid (5-ASA) and azathioprine (2 mg/kg/day).	Po. cyclosporine initial dose of 5 mg/kg/day in two divided doses. The dose was increased to 150–250 ng/mL. This was achieved in most cases with a dose of 7–8 mg/kg/day in 3–4 days.	87% 28/32 patients responded quickly to treatment (clinically and laboratory improve)	12.5% 4/32 did not respond and underwent colectomy.	Treatment with oral CyA altered the course of UC in 28/32 (87%) of patients; of the 28 patients that responded to CyA, 5 underwent later elective colectomy. Overall, in 72% of patients, colectomy was avoided.	80% 28%—9/32 patients required total colectomy. 35%—7/20 of the patients with steroid dependent UC and 17%—2/12 with steroid resistant UC underwent colectomy.	Severe headaches and paresthesia (*n* = 1), mild headaches (*n* = 4)
Piotr Socha, [20]	observational retrospective	10 (of 23)	2.75–18.5 years	steroids, azathioprine	cyclosporine dose to obtain levels (serum concentration > 100 ng/mL and <200 ng/mL).	60% 11/21			100% 10/10 patients underwent surgery	hirsutism (*n* = 1), gingival hypertrophy (*n* = 1)

**Table 3:** Summary of studies combining ustekinumab and cyclosporine.

Author	No. of Patients	Age	Previous Unsuccessful Treatment	Current Treatment Used	Time to Achieve Remission	Avoidance of Colectomy [%].	Serious Adverse Events
Ganzleben I, [42]	1	33 men	adalimumab, infliksimab, wedolizumab, cyclosporine in combination with azatiopryną and merkaptopuryną, tofacitinibem, prednizolon—No clinical improvement or discontinued due to side effects.	Cyclosporine iv 2 mg/kg, On day 6 of cyclosporine treatment 390 mg iv. Ustekinumab.	1 patient on day 62 of treatment partial Mayo score: 0	100%	0%
GC Shaffer, Seth R. [45]	2	21 men 20 women	1 pacjent—mesalamina, wedolizumab, sterydotenapia 2 pacjent—adalimumab, tofacitinib, sterydotenapia, wedolizumub	1 patient—cyclosporine iv. 3 mg/kg, 4th day of therapy ustekinumab 390 mg iv. 2 patient—cyclosporine po and ustekinumab 260 mg iv.	1 patient—on day 4 of cyclosporine treatment discharged home; 2 patient—improved after 2 weeks of treatment.	1—100% 2—100%	1—0% 2—0%
Vitali F [44]	11 (5 men and 6 women)	42.3 (37–66)	11 patients—hydrocortisone and prednisolone, 10—infliximab 7—vedolizumab 5—tofacitinib	11—cyclosporine iv. 2 mg/kg, after 5 days cyclosporine po. ustekinumab iv. 6 mg/kg body weight was started an average of 3.2 (1–8) weeks after initiation of successful intravenous cyclosporine induction therapy. Subcutaneous maintenance therapy with ustekinumab (90 mg) was given every 6–8 weeks	Clinical response—6 patients at week 16, Clinical remission without steroids—5 patients at week 48, Endoscopic remission—5 patients at week 16, discontinued therapy due to ineffectiveness—3	2/11 patients had to undergo colectomy (average 4.5 months, range 3–6) 18.18%	5 of 11 (45%) patients (paresthesias, muscle aches, headaches, nausea, hair loss), which were attributed to cyclosporine
Pauline Veyrard [43]	10 (8 women, 2 men)	32 (25.2–37.8)	10 patients—steroid therapy (0.8–1 mg/kg) 9—infliximab 8—vedolizumab	cyclosporine iv. 2 mg/kg/d, followed by po. ustekinumab (6 mg/kg) followed by 90 mg subcutaneously every 8 weeks.	The median partial Mayo score and C-reactive protein levels decreased significantly after 6 months: 10.6 versus 1.0 (*p* = 0.005) and 17.5 (IQR, 4.1–38.8) mg/dL versus 2.3 (IQR, 1.7–3.2) mg/dL, respectively (*p* = 0.02). Clinical response and remission at 6 months: 90%	After 6 months—100%	1 case of alopecia—treatment continued

## Data Availability

Not applicable.
